# Editorial: Core–Shell Nanostructures for Energy Storage and Conversion

**DOI:** 10.3390/nano13030589

**Published:** 2023-02-01

**Authors:** Zhipeng Sun, Ruiying Wang

**Affiliations:** 1 School of Materials and Energy, Guangdong University of Technology, Guangzhou 510006, China; 2 State Key Laboratory of Chemistry and Utilization of Carbon-Based Energy Resources, College of Chemistry, Xinjiang University, Urumqi 830017, China

Owing to their special physical and chemical properties, nanomaterials with core–shell structures have been extensively synthesized and widely studied in the field of energy storage and conversion. The goal of energy storage and conversion will be facilitated by designing and fabricating core–shell structural nanocomposites that possess many promising virtues. For instance, the shell supported by the core guarantees the specific surface architecture relying on the porosity, surface area, etc., resulting in outstanding electrochemical performance. Moreover, the synergistic interactions between the shell and core are beneficial to realize advanced electrochemical properties.

Here, we hope that recent developments in the research of various types of core–shell structure nanomaterials in the field of energy storage and conversion will be communicated well in this Special Issue. This Special Issue comprises five articles. McVey et al. synthesized a core–shell Cd_3_P_2_/Zn_3_P_2_ composite and studied its structural (morphology, crystallinity, shell diameter), chemical (composition of core, shell, and ligand sphere), and optical properties (absorbance, steady-state and time-resolved emission, quantum yield, and air stability) [1]. Shi et al. prepared a MOF-derived NiSe@C composite exhibiting excellent sodium-ion storage properties [2]. Song et al. fabricated a Ni_2_P@Fe_2_P core–shell nanostructure with outperforming OER performance through the chemical transformation of rationally designed Ni-MOF composite nanosheets [3]. Song et al. fabricated a unique core–shell structure integrating CoMoO_4_ as support frameworks coated with 2D γ-FeOOH nanosheets on the surface. By involving CoMoO_4_, the electrochemically active surface area can be significantly enhanced [4]. Hwang et al. prepared Si@C nanoparticles through a solvent-assisted wet coating method, achieving excellent specific capacity and capacity retention [5].

This Special Issue will promote developments and innovative ideas through fruitful discussions among researchers in the field of nanomaterials and energy.
